# Retraction: Construction and optimization of inventory management system via cloud-edge collaborative computing in supply chain environment in the Internet of Things era

**DOI:** 10.1371/journal.pone.0291318

**Published:** 2023-09-06

**Authors:** The PLOS ONE Editors

The *PLOS ONE* Editors retract this article [1] because it was identified as one of a series of submissions for which we have concerns about peer review integrity and similarities across articles. These concerns call into question the validity and provenance of the reported results. We regret that the issues were not identified prior to the article’s publication.

The author either did not respond directly or could not be reached.

## References

[1] Ran H (2021) Construction and optimization of inventory management system via cloud-edge collaborative computing in supply chain environment in the Internet of Things era. PLoS ONE 16(11): e0259284. 10.1371/journal.pone.0259284 34731183 PMC8565765

